# Systematic review of health risk assessment in Africa’s bushmeat trade: Are there any risks assessed?

**DOI:** 10.1371/journal.pntd.0014308

**Published:** 2026-05-18

**Authors:** Claude Vianney Amougou, Alain Didier Missoup, Maurice Tindo, Philippe Gaubert

**Affiliations:** 1 Zoology Unit, Laboratory of Biology and Physiology of Animal Organisms, Faculty of Sciences, University of Douala, Douala, Cameroon; 2 Centre de Recherche sur la Biodiversité et l’Environnement, Université de Toulouse, CNRS, IRD, Toulouse INP, Toulouse, France; 3 CIIMAR/CIMAR, Interdisciplinary Centre of Marine and Environmental Research, University of Porto, Matosinhos, Portugal; 4 Laboratoire d’Ecologie Appliquée, Faculté des Sciences Agronomiques, Université d’Abomey-Calavi, Calavi, Benin; Colorado State University, UNITED STATES OF AMERICA

## Abstract

**Background:**

The bushmeat trade in tropical Africa represents a major route for zoonotic disease emergence. Yet, the extent to which health risks have been quantitatively assessed remains unclear. Therefore, our study aimed at systematically reviewing health risk assessments conducted in the African bushmeat trade, and identifying methodological patterns and research gaps.

**Methodology/principal findings:**

Following PRISMA guidelines, we searched Web of Science and Google Scholar (to November 2024) using multilingual Boolean queries. Eligible studies included any research assessing health, zoonotic, or food-borne risks in bushmeat supply chains across Africa. Two co-authors independently cross-validated c. 23% of extracted data. Descriptive statistics and generalized linear models were used to explore publication patterns and predictors of research output. From 449 records finally identified, 129 met inclusion criteria. Ethnobiological and public health surveillance approaches dominated (41.1% each), while epidemiological studies were scarce. Most publications appeared after 2017, mainly from Cameroon, Nigeria, and the Democratic Republic of Congo, with epidemic occurrence significantly predicting national research output. Interviews were the most frequent method (44.8%), while pathogen detection occurred in 40.3% of studies, identifying 66 confirmed human pathogens (23 viruses, 19 bacteria, 24 parasites). More than 88% of studies did not report survey effort, and none implemented a formal quantitative health risk assessment.

**Conclusions/significance:**

Quantitative health risk assessment in the African bushmeat trade remains unattainable due to scarce data on pathogen prevalence, exposure, and host–pathogen interactions. Only formal recognition and state-regulated management of the trade—incorporating molecular surveillance, host-pathogen ecological data, and supply-chain mapping within a One Health framework—will enable reliable risk quantification.

## Author summary

The bushmeat trade in tropical Africa is a recognized pathway for zoonotic disease emergence, yet the health risks associated with this trade remain poorly quantified. We conducted a systematic review of studies assessing health risks in bushmeat value chains across Africa, following PRISMA guidelines. Of 449 records identified, 129 studies met inclusion criteria. Most research employed ethnobiological or public health surveillance approaches, while epidemiological studies were rare. Research output was concentrated in Cameroon, Nigeria, and the Democratic Republic of Congo, with epidemic events driving survey effort. Interviews were the predominant method, and direct pathogen detection occurred in less than half of studies, identifying 66 confirmed human pathogens, including viruses, bacteria, and parasites. Notably, over 88% of studies did not report survey effort, and none performed a formal quantitative health risk assessment. Our findings highlight that robust risk quantification is currently unfeasible due to limited data on pathogen prevalence, human exposure, and host–pathogen interactions. To enable reliable assessment, the bushmeat trade must be formally recognized and managed by national authorities, integrating molecular surveillance, ecological data, and supply-chain mapping within a One Health framework. This approach is essential for anticipating zoonotic spillovers and guiding evidence-based public health interventions.

## Introduction

While a portion of the wildlife trade operates legally under international frameworks such as the Convention on International Trade in Endangered Species of Wild Fauna and Flora (CITES), a substantial share remains illicit. The illegal wildlife trade (IWT)—involving the trafficking of live or dead wild species and their derivatives—constitutes a highly profitable shadow economy that sustains trafficking networks across multiple geographic scales [1,2]. The IWT has long been recognized by both scientific and inter-governmental sectors as a significant driver of zoonotic spillover risks [3,4], facilitating an estimated one billion human–wildlife interactions each year [5]. These risks are further exacerbated by economic globalization [6], as starkly demonstrated by the COVID-19 pandemic [7], which heightened global awareness of the public health threats linked to wildlife trafficking. In response, there have been increasing calls to adopt an integrated “One Health” approach to mitigate the risk of future zoonotic outbreaks (e.g., [8]).

The bushmeat trade (BT) is an informal, largely unregulated, and predominantly illegal activity occurring across tropical regions, primarily targeting terrestrial vertebrates [9]. In Africa, the BT represents a long-standing cultural tradition and serves as a critical source of both animal protein and income for many rural communities [10]. In the Congo Basin alone, around 4.5 million tons of bushmeat are harvested annually, placing unsustainable pressure on wildlife populations [11,12]. Beyond its ecological impacts, the BT poses serious public health risks, functioning as a vector for zoonotic pathogens such as Ebola, Mpox, and HIV [13–15]. The growing encroachment of human activities, including deforestation and habitat fragmentation, further elevates the risk of zoonotic spillovers, underscoring the urgent need to address the health hazards associated with bushmeat consumption in Africa [16,17].

Although more pathogens linked to bushmeat are now being studied, the specific practices that increase the risk of transmission are still not well understood or systematically mapped [18–20]. Several studies have identified risky behaviors that can lead to cross-species transmission, including contact with contaminated materials and blood during hunting, transportation and sale of bushmeat [21–23]. Long-distance bushmeat transport increases the chances of zoonotic spillovers, especially for pathogens that can survive in changing environments [24,25]. Direct spillover can also happen through bites, scratches, or cuts during hunting or butchering [26,27].

Practices at risk are often driven by deep-rooted economic, cultural, social, and dietary factors (e.g., [19,28,29]), and are usually more common where people face poverty or food insecurity [30,31]. Moreover, people involved in the bushmeat supply chain—such as hunters, butchers, sellers, and consumers—do not all have the same understanding of health risks. While some, especially urban sellers, recognize the link between bushmeat and zoonotic pandemics (e.g., [26,32], others have limited or unclear perceptions. This gap is often due to limited access to health services and weak disease reporting systems in many regions [19]. Public health messaging may also be perceived as exaggerated, and ultimately ignored, while the use of mitigation measures such as wearing gloves, thorough cooking, or face masks remains rare, particularly in informal markets [33,34].

In this study, we focus on the notion of health risk assessment within the context of Africa’s BT. Although numerous studies have addressed zoonotic risks associated with the BT in Africa (see above), it remains unclear how such risks have been assessed, and to what extent existing assessments can inform national and regional zoonotic spillover prevention strategies. Health risk refers to the probability that exposure to specific agents or conditions will negatively affect human health (https://newsinhealth.nih.gov/2016/10/understanding-health-risks). In the BT, health risk can thus be defined as the probability of zoonotic spillovers or food-borne infections arising from human contact with animal fluids, carcasses, or meat consumption [35]. Such risk is conditioned by multiple factors, including the diversity and volume of species traded, the structure of supply networks, pathogen prevalence, and individual susceptibility to infection [36,37].

Here, we present a systematic review of health risk assessments conducted in the context of the BT in tropical Africa. Specifically, we identify global trends in health surveys on bushmeat consumption and trade, and characterize the experimental designs used to quantify associated health risks. On this basis, we discuss the feasibility of quantifying health risks in the African bushmeat context and propose pathways to strengthen health risk assessment frameworks along BT networks in tropical Africa.

## Methods

### Database on health risk assessment of the bushmeat trade in African rainforests

**Fig 1:**
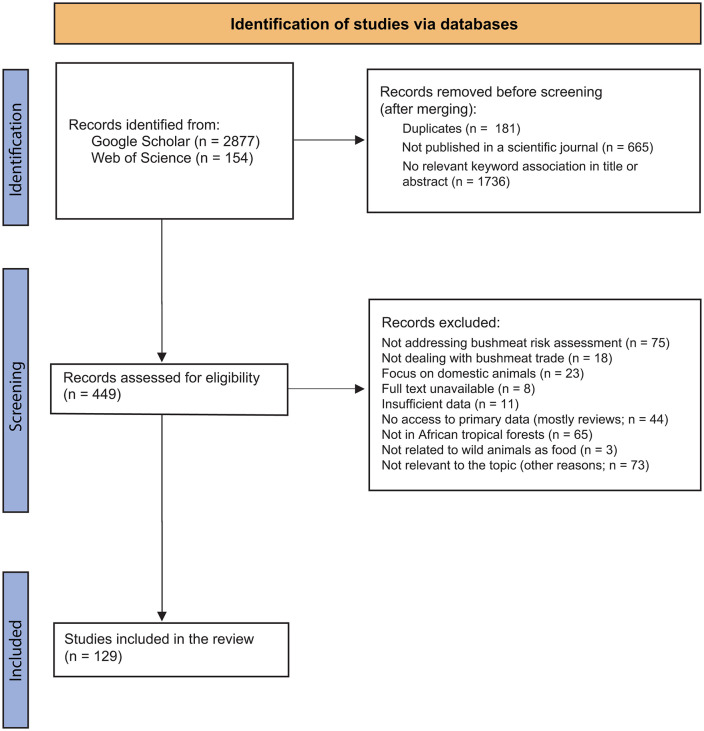
PRISMA flow diagram illustrating the article search and screening process of the systematic review on health risk assessments related to the bushmeat in African tropical rainforests.

This study was conducted in accordance with the Preferred Reporting Items for Systematic Reviews and Meta-Analyses (PRISMA) guidelines and procedures [38] (Fig 1, S1 Table). We focused on articles published in scientific journals, to assess global trends in health surveys related to the BT and investigate how scientists assess and quantify the health risks associated with the consumption, handling, and trade of bushmeat. We used full-text peer-reviewed articles—available through open access—that refer to ‘wild meat’ or ‘bushmeat’ in relation to health risk assessment. Review articles were included in the database if only they contained secondary data, new data or if we were unable to access the primary data. Only studies conducted within African tropical rainforests were considered, here including the Congolian region (as defined in [39]), together with remnant forest patches within the Sudanian region (e.g., Senegal and Guinea-Bissau; see [40]). This geographic restriction was applied to focus on areas where the BT is particularly pronounced and has been identified as a significant threat to human health [41,42]. This also ensures that climate and vegetation contexts are similar across the study area; thereby reducing the possibility of bias due to data heterogeneity, since wildlife handling practices, bushmeat species and therefore the associated health risks are likely to be similar.

The literature search was conducted between October 2023 and November 2024, with no restriction on publication years. Boolean search terms were used to identify literature from two databases; namely the Web of Science and Google Scholar. The reason being that the Web of Science generally contains high-quality studies, which are selected through rigorous indexing and precise filtering tools. On the other hand, Google Scholar provides access to a wider range of literature, particularly local and grey literature, which are often absent from traditional databases. Combining the two databases enables us to access the vast majority of available information on the subject. The search was designed such as not to exclude articles written in French, English, Spanish or Portuguese. In the Web of Science (WoS), the Boolean search string ((health risks OR health hazards OR health threats OR risks OR health OR zoonos* OR pathogen*) AND (bushmeat markets OR venison OR wild meat markets OR bushmeat OR wild meat) AND (Africa OR sub-Saharan Africa)) was used. The search returned 154 articles, of which only those containing “health risks” (or “health hazards”, “health threats” or “pathogens”), “market” and “bushmeat” (or wild meat/ wildmeat) in their title and/or abstract were included in the database (N = 69). The same search string was used in Google Scholar, but given the great number of returned articles (N = 5050), we narrowed down the search with the following string: ((health OR sanitary risk*) AND zoonoses* AND (bushmeat OR “wild meat”) AND market* AND Africa), to obtain 2877 articles. After merging the two databases, duplicate articles were manually removed (N = 181), references not being articles published in scientific journals (e.g., books, reports; N = 665) and articles not containing “bushmeat” (or wild meat or venison) and “health” in the title or the abstract were discarded (N = 1736) to form an initial reading list of 449 records. Studies conducted outside bushmeat markets (e.g., in a protected area, a rural community or a hospital) were excluded if not referring to wild animals as meat or as being intended for human consumption. Specifically, rural community studies were included when they directly addressed bushmeat-related activities. Studies focusing on domestic animals were also excluded. On this basis and from the criteria previously described, we excluded reviews and articles for which we did not have access to primary data, studies that did not provide sufficient data or did not address bushmeat risk assessment, and those conducted outside the targeted study area (see S2 Table for full details). The final list of publications consisted of 129 articles (compiled on 22/11/2024; S3 Table and S1 Appendix). Two co-authors (ADM and PG) independently reviewed 15 randomly chosen articles (23.3% of the total) of the list for cross-check validation.

In accordance with PRISMA guidelines [38], risk of bias was considered at both the review and study levels. At the review level, potential publication bias may result from the reliance on Web of Science and Google Scholar, which, despite broad coverage, may incompletely capture grey or non-indexed literature. Although multilingual search terms were used, language and indexing biases cannot be entirely excluded. In addition, the screening strategy based on titles and abstracts may have led to the exclusion of relevant studies using inconsistent terminology.

At the study level, no standardized risk-of-bias assessment tool was applied due to the substantial heterogeneity in study designs, objectives, and outcome measures. However, several sources of bias were identified. Selection bias may arise from the geographic restriction to tropical African forest bioregions and from study-specific sampling strategies that were often poorly described. Reporting bias was substantial, as most studies did not provide key methodological information (e.g., survey effort, sampling duration), limiting the assessment of internal validity. Measurement bias is also likely, given the predominance of self-reported data (e.g., interviews) and the variability in laboratory and observational methods used to identify pathogens or risk practices.

Missing data were not imputed; instead, analyses were conducted using available data only. When key methodological or outcome information was absent, this was recorded during data extraction and considered in the interpretation of results. Data extraction bias was mitigated through independent cross-validation of a subset of studies (23.3%) by two co-authors. Nevertheless, the high degree of methodological heterogeneity precluded quantitative synthesis and formal bias comparison across studies, and may affect the robustness and generalizability of the reported trends.

Previous reviews have discussed health risks associated with bushmeat in tropical Africa, either by highlighting the ecological and epidemiological factors of these risks [35,42,43], analyzing the practices at risks of stakeholders in the bushmeat supply chain [44], or examining the potential for exposure to and/or contamination by zoonotic pathogens [45–47]. To our knowledge, our review is the sole to critically scrutinize the assessment of health risks related to the bushmeat trade in African tropical rainforests.

### Data extraction

Articles were categorised into three main research areas, including public health surveillance (collection of data on pathogens and/or biomonitoring in relation to health), ethnobiology (study of human behaviour, practices at risk and perceptions in relation to bushmeat handling) and epidemiology (means of transmission and/or causal links between certain factors / practices and zoonotic diseases).

We collected and analyzed data to identify global trends in health surveys on bushmeat consumption and trade, and to characterize the experimental designs used in these studies. We focused on temporal indicators such as year of publication, study period, time lag between (i) survey and publication years and (ii) that of epidemic outbreak (when applicable) and publication. We also collected geographic information on the country, location of study site, type of study site, and study scale.

More information of the context and methodology of the studies were gathered, including survey effort, survey targets, main objective(s), and risk assessment approach. Based on the various components of health risk assessment (identify and assess the hazard, estimate/evaluate exposure; [48]), we scored whether the studies had conducted an actual estimate of the health risks, through the establishment of a probability, a score or any other conclusion in risk weighting. We also collected information on (i) whether each study was general or focused on specific pathogens, and (ii) the pathogens targeted. We classified the pathogenicity of the microorganisms based on databases from public health sources, including the Centers for Disease Control and Prevention [49,50], the World Health Organization [51], and NCBI (https://www.ncbi.nlm.nih.gov/pathogens/organisms/), together with reference peer-reviewed literature [52]. Specific disease associations were verified for each taxon.

### Data analysis

Descriptive statistics and graphical outputs were conducted in Microsoft Excel (Microsoft 365) using dynamic cross-tabulations, and RStudio version 4.3.1 [53] with *ggplot2*, *reshape2, tidyverse* and *rnaturalearth* packages. We used *AER* package to run a General Linear Model (GLM) based on the quasi-poisson regression model to quantify the relationship between the number of scientific publications (dependent variable) and a series of country-specific predictor variables. These included GDP (see [54,55]), urbanization rate (considered a catalyst for the spread of zoonoses; [56]), together with forest cover and the occurrence of major epidemics (i.e., large scale, rapidly spreading outbreaks with significant health, social, or economic impact), both of which may increase research prioritization in impacted countries [57,58]). GDP and urbanization rate data were obtained from World Bank (https://www.worldbank.org; year 2022), while forest cover percentages were obtained from UN Food and Agriculture Organization (FAO, https://ourworldindata.org/forest-area; year 2020). The presence or absence of zoonotic outbreaks (Ebola, Monkeypox, Lassa fever, and Marburg) was determined through searches in the Google database, supplemented with information from Pasteur Institute (https://www.pasteur.fr/fr/centre-medical/fiches-maladies) and World Health Organization (https://www.who.int/fr/emergencies/disease-outbreak-news).

## Results

Among the three research fields investigating zoonotic risks associated with bushmeat in African tropical rainforests, ethnobiology and public health surveillance were the main contributors, accounting for 41.1% of the scientific output, respectively, while epidemiology represented 17.8%. Four of the 94 scientific journals involved accounted for approximately 20.2% of the articles: *EcoHealth* (6.9%), *Emerging Infectious Diseases* (4.7%), *PLoS ONE* (4.7%), and *PLoS Neglected Tropical Diseases* (3.9%).

**Fig 2:**
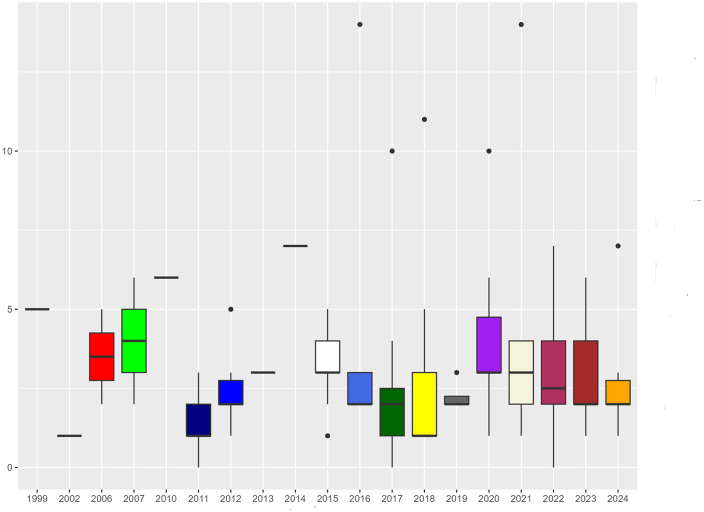
Time lag between survey and publication year of risk assessment studies on the bushmeat trade in African tropical rainforests. Time lag is expressed in years.

Most scientific articles on the topic (63.5%) were published from 2017. The majority of articles (70.7%) was conducted within one year of survey, with a maximum duration of 15 years. The data collection period was not reported in 17.8% of the cases. The time lag between the end of the survey period and the publication ranged from 0 to 14 years, with the majority of articles (50.9%) being published within two years (Fig 2). Only 24 studies (18.6%) were reported to have been conducted in response to a zoonotic outbreak. The time lag between the occurrence of the epidemic in question and the publication of the study ranged from a few months to 14 years (mean = 3.8 yrs; SD = 3.4).

**Fig 3:**
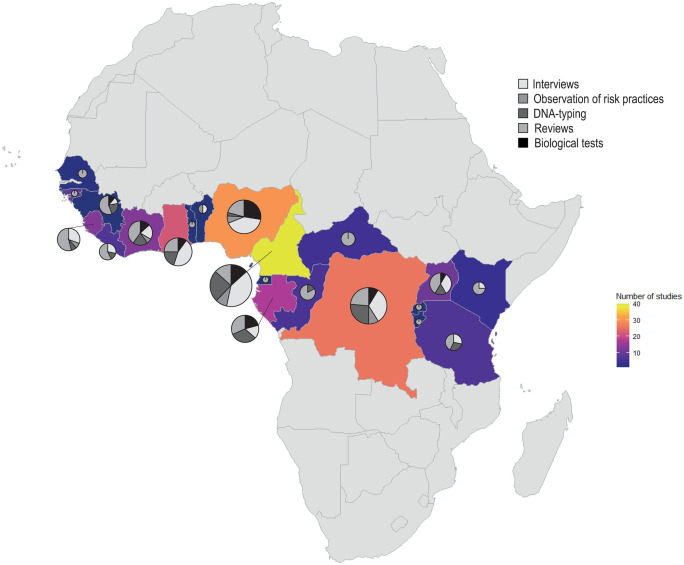
Per-country number of scientific publications on health risk assessment in African tropical rainforests together with assessment methods. Pie charts show the proportion of assessment methods used in each country (e.g., pie charts in Togo and Cameroon represent one and 40 articles, respectively). Base map layer obtained from Natural Earth (public domain data), via the *rnaturalearth* R package. Country boundaries sourced from: https://www.naturalearthdata.com/downloads/50m-cultural-vectors/50m-admin-0-countries/. License information available at: https://www.naturalearthdata.com/about/terms-of-use/.

**Fig 4:**
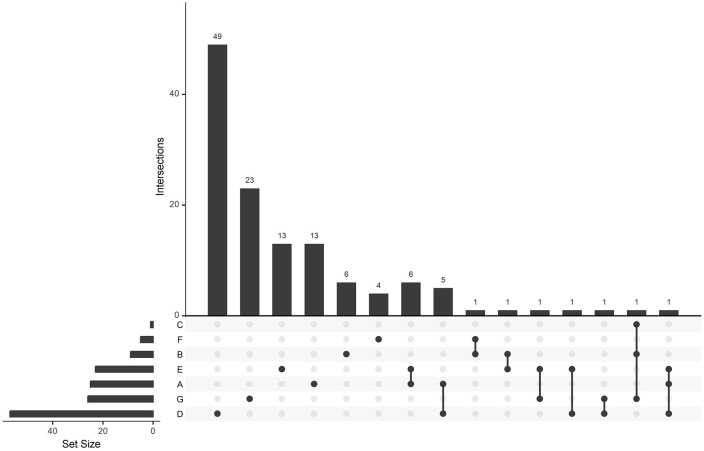
Type of study sites targeted by health risk assessment surveys related to the bushmeat trade in African tropical rainforests. A: Forest and protected areas; B: Hospitals; C: Households and schools; D: Rural communities; E: Rural markets; F: Urban communities; G: Urban markets.

Cameroon (n = 40), Nigeria (30), and the Democratic Republic of Congo (26) were the most studied countries (Fig 3). Most of the studies (88.4%) were deployed at the national level, while only 5.4% investigated an international scale. Study sites varied but were predominantly represented by rural communities (~36.5%) and urban markets (~19.9%). Approximately 76.2% of the articles focused on a single type of study site (Fig 4).

**Fig 5:**
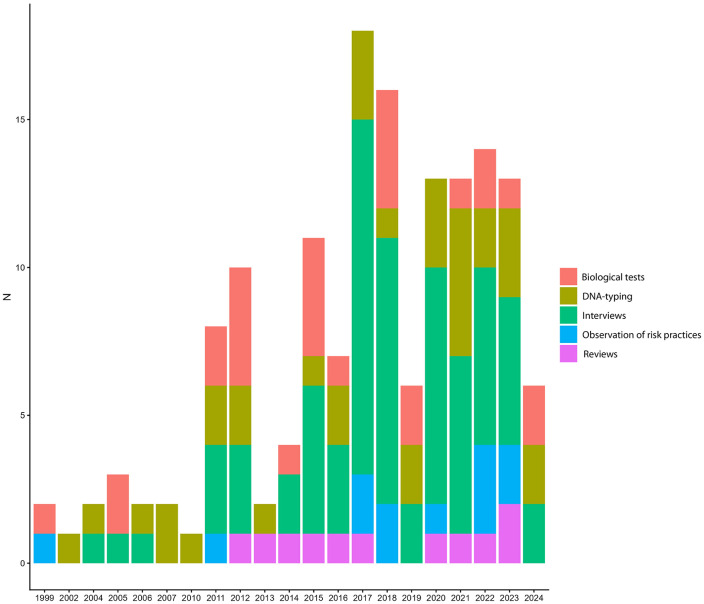
Temporal distribution of survey methods used in health risk assessment related to the bushmeat trade in African tropical rainforests.

Approximately 83.7% of the studies did not report the survey effort (number of days over which data collection occurred). When the information was given, data collection was deployed from 3 to 2670 days (mean = 178.9 days; SD = 574.5). Health risk assessment was conducted on humans (56.1%) and on the bushmeat species (39.4%), while only 4.5% considered both humans and bushmeat. Most studies relied on a single assessment method (80.6%). Interviews were the predominant approach (44.8%), with a marked increase from 2017 (Fig 5). The use of other approaches was moderate, including biological tests (17.5%), article reviews (7.2%), observation of risk practices (7.8%) and DNA-typing (22.7%).

All the scientific studies focused on identifying potential risks or hazards linked to bushmeat without addressing the likelihood of their occurrence. This was done either through observational studies on human behavior or laboratory-based epidemiological research. Eventually, 53.5% of the studies did not propose any recommendations.

GLM analysis suggests that the presence of major epidemics and, to a lesser extent (marginally significant effect), GDP were the variables significantly explaining the number of health risk surveys conducted among countries (Table 1).

**Table 1:** Generalized linear model assessing the influence of GDP, forest cover, urbanization rate, and major epidemics on the number of bushmeat health risk surveys conducted in African tropical rainforests.

	Estimate	Std. Error	t-value	Pr(>|t|)
(Intercept)	3.075e^-01^	7.800e^-01^	0.394	0.6986
GDP	2.562e^-12^	1.291e^-12^	1.984	0.0647
Forest cover	7.538e^-03^	1.123e^-02^	0.671	0.5115
Urbanisation	3.485e-03	1.773e^-02^	0.196	0.8467
Major epidemics	1.573e + 00	6.856e^-01^	2.294	0.0357

The microbes and other organisms identified in the bushmeat included 36 viruses, 38 bacteria and 38 parasites (Table 2). Our study revealed that 66 of the identified microbes and parasites contain taxa known to be pathogens in humans (23 viruses, 19 bacteria and 24 parasites). Another 11 viruses and 10 bacteria are opportunistic or uncertain human pathogens. The other taxa reported are host pathogens not known to cause human disease or commensal microbes.

**Table 2:** List of the microbes and parasites identified from bushmeat surveys in African tropical rainforests.

Virus	Bacteria	Parasites
Alphacoronavirus+++	*Achromobacter++*	*Ancylostoma+++*
Alphatorquevirus++	*Anaplasma+++*	*Armillifer+++*
Betacoronavirus+++	*Bacillus+++*	*Ascaris+++*
Betatorquevirus++	Bacteroidota++	*Babesia+++*
BtKY56/ BtKY55++	*Brucella+++*	*Balantidium+++*
Cytomegalovirus++	*Campylobacter+++*	*Bertiella+++*
Deltaretrovirus+++	*Candidatus*	*Capillaria+++*
Duvenhage virus++	Chloroflexota	*Cryptosporidium+++*
Ebola virus+++	*Clostridium+++*	*Dicrocoelium+++*
Enterovirus+++	*Coxiella+++*	*Eimeria*
Gemykibivirus++	Cyanobacteria	*Endolimax*
Henipavirus+++	*Deinococcota*	*Entamoeba+++*
Human immunodeficiency virus 1 and 2+++	*Ehrlichia+++*	*Enterobius+++*
KY22/2006++	*Escherichia+++*	*Fasciola+++*
Lagos bat lyssavirus+++	Fibrobacteria	*Giardia+++*
Lassa virus+++	*Klebsiella+++*	*Globocephalus*
Lentivirus+++	*Lactobacillus++*	*Haemaphysalis+++*
Lymphocryptovirus+++	*Leptospira+++*	*Haemonchus*
Lymphocytic choriomeningitis virus+++	*Midichloria*	*Heligmosomoides*
Marburg virus+++	*Mycobacterium+++*	*Hyalomma+++*
Mastadenovirus+++	Planctomycetes	*Hymenolepis+++*
Monkeypox virus+++	Proteobacteria	*Iodamoeba*
Orthohepadnavirus+++	*Proteus++*	*Mammomonogamus+++*
Orthomyxoviridae+++	*Providencia++*	*Metastrongylus*
Orthopoxvirus+++	*Pseudomonas++*	*Moniezia*
Pegivirus++	*Ralstonia++*	*Moniliformis*
Picobirnavirus++	*Rickettsia+++*	*Oesophagostomum+++*
Rabies lyssavirus+++	Saccharibacteria++	*Paramphistomum+++*
Rift Valley fever virus+++	*Salmonella+++*	*Paraspidodera*
Rotavirus+++	Shiga toxin-producing *Escherichia coli*+++	*Protostrongylus*
Simian & Human T-lymphotropic viruses+++	*Shigella+++*	*Schistosoma+++*
Simian Foamy Virus++	*Spirochaetota+++*	*Spirura*
Simian Immunodeficiency Virus	*Staphylococcus+++*	*Strongyloides+++*
Spumavirus ++	*Stenotrophomonas++*	*Strongylus+++*
Tetraparvovirus+++	*Streptococcus +++*	*Taenia+++*
Tibrovirus++	*Synergistota++*	*Toxocara+++*
	Tenericutes+++	*Trichostrongylus*
	Verrucomicrobiota	*Trichuris*

+++ confirmed human pathogen; ++ opportunistic or uncertain human pathogen. The other microbes and parasites are either commensal or pathogens specific to non-human animals.

Among the hundred vertebrate species reported as hosts (S4 Table), mammals were the most frequent, while 12.7% of animals could not be specifically identified. Pathogens were screened in only 55 articles (40.3%). A majority of authors (92.5%) focused exclusively on the identification of a single group of pathogens, with 56.9% of these studies concentrating on viral diseases.

## Discussion

### Global trends in health surveys on bushmeat consumption and trade

The first objective of our review was to examine global trends in health risk assessments conducted by scientists on bushmeat consumption in African tropical rainforests over the past 25 years. Our study revealed a co-dominance of investigations based on ethnobiological and public health surveillance approaches. Because understanding the practices and behaviors of local populations is key to evaluating the associated health risks [59], especially given that the risk of zoonotic spillovers is strongly influenced by cultural practices and close contact with wildlife [58], ethnobiology stands as a major research domain in health risk assessments. Additionally, the frequent use of public health surveillance aligns with the global recognition of bushmeat as a significant source of zoonotic risks [35,42,43]. By accumulating long-term data, public health surveillance can provide a valuable tool for monitoring zoonotic spillover risks in human populations [60]. In contrast, relatively few studies have adopted an epidemiological framework, despite its effectiveness in quantifying health risks [42]. Overall, this global trend in health risk assessments underscores the prevailing lack of an interdisciplinary framework to comprehensively address the health risks associated with the BT in African tropical rainforests, despite repeated calls for more integrative approaches, such as One Health [36,61,62].

Approximately 20% of the scientific surveys were published in four journals—two specializing in emerging diseases, one in global health sciences, and one with a broad, inclusive scope. Alongside the other 90 journals, this publication scheme shall contribute to the widespread dissemination of multidisciplinary findings to a diverse audience. Most research on bushmeat health surveys has been published since 2017, possibly reflecting a growing interest in the topic—likely influenced by recent zoonotic outbreaks [58]. This interest may be particularly relevant in the context of emerging epidemics and pandemics such as Ebola and COVID-19 [63,64]. However, the increase in publications does not necessarily indicate a reactive investment in understanding and mitigating health risks related to bushmeat consumption. Instead, it should be considered alongside the overall growth rate of scientific publications in the Life Sciences, which averages 5.07% per year, with a doubling time of 14.0 years [65].

Cameroon, Nigeria, and DR Congo accounted for approximately three-quarters of the bushmeat health surveys, aligning with Groom et al. [54]’s findings on the global distribution of bushmeat survey efforts across African tropical rainforests. These countries have experienced significant epidemic outbreaks [66,67] and rank among the highest GDPs in the study region—both factors that correlate positively with the global frequency of bushmeat surveys. Additionally, they serve as key hubs for national and international bushmeat trade [68–70], including the trafficking of pangolins [54,71], likely promoting the prioritisation of health risk surveys.

Most studies have been conducted at the national level, with only 5.4% examining the international scale. National-scale surveys were typically limited to a single site, likely failing to capture the complex, interconnected network of the BT. The scarcity of studies investigating international trade reflects a broader issue of inadequate scale design in bushmeat research [54]. This shortfall is partially attributed to a global lack of funding (e.g., [72]) but also to the insufficient prioritization of bushmeat-related health risks in the northern hemisphere [73]. Notably, the large volumes of bushmeat entering Europe and North America each year (e.g., [70,74,75]) pose a significant yet under-assessed threat to public health in these regions [35,76].

The predominant targets of the health surveys were rural communities, and to a lesser extent, urban bushmeat markets. Due to their proximity to and involvement in hunting and bushmeat trade-related activities, rural populations face a heightened risk of direct contact with bushmeat species, particularly during fresh carcass processing—a critical high-risk phase in the bushmeat trade chain (e.g., [77]). The observed similarities between human and wildlife viromes further underscore the likelihood of such interactions, especially with non-human primates [27,78]. Urban bushmeat markets, on the other hand, increase the frequency and diversity of human-animal interactions, potentially creating multiple opportunities for zoonotic disease emergence and spread [79]. However, the health risks across the entire bushmeat trade chain—from hunting to final consumption—remain insufficiently assessed and properly scaled (see [30, 80]). Notably, as the trade chain progresses, the time elapsed since the animal’s death increases, which may reduce the risk of zoonotic transmission, particularly for RNA viruses, despite their persistent detectability [81,82], while increasing the risk of food-borne disease spillover [30].

Our study identified a codominance of pathogenic taxa to humans among viruses, bacteria and parasites (23, 19 and 24 genera, respectively). These findings align with a recent review by Moloney et al. [83] on the pathogenic spectrum detected in wild meat. More than half of the pathogens reported in bushmeat samples are involved in zoonotic spillovers –or likely cause diseases– in humans. Such substantial pathogen diversity highlights the significant potential of bushmeat as a source of emerging infectious diseases [23,43]. Most surveys focused on viral diseases, as viruses are highly transmissible, evolve rapidly, and often have limited therapeutic options, making them significant epidemic and pandemic threats [84]. Health risk assessments of the bushmeat trade have primarily targeted major viral agents, including the Ebola virus [85–87], various strains of simian immunodeficiency viruses [88], and the Mpox virus [89]. However, we observed a striking absence of broad-spectrum pathogen surveillance in bushmeat, which could hinder the proactive identification of emerging threats, including the prospective search for Disease X [83].

Among the approximately 100 vertebrate species identified as pathogen hosts in this review, the majority were mammals, aligning with previous findings on bushmeat diversity and research focus (e.g., [90]). Mammals are the primary target of health risk surveillance in the bushmeat trade because (i) they constitute the dominant taxonomic group in markets [54] and (ii) their phylogenetic proximity to humans increases the likelihood of zoonotic transmission [91]. In contrast, other vertebrate taxa present in the bushmeat trade, such as birds, reptiles and amphibians, remain largely under-investigated despite their known potential for zoonotic transmission [92–94]. A key limitation of bushmeat surveys is the frequent uncertainty in taxonomic identification of host species [54]. Our review revealed that health risk assessments of the bushmeat trade were also affected by inaccuracies in taxonomic identification, potentially obscuring host-pathogen relationships and hindering our understanding of zoonotic spillovers. To address these challenges, bushmeat health surveys could benefit from routinely integrating DNA-based methods for more precise host identification (e.g., [95,96]). This recommendation also extends to the “untargeted” screening of pathogen communities (see [83,97]), as our review found that only about one-quarter of health risk surveys incorporated molecular tools.

### Experimental design in bushmeat health surveys and a blatant lack of zoonotic risk quantification

Our systematic review revealed significant flaws in the experimental design of health surveys targeting the bushmeat trade in African tropical rainforests. Nearly one-fourth of the studies failed to report the data collection period, while approximately 83.7% did not disclose survey effort (site.days). The lack of transparency in temporal and survey efforts undermines the reproducibility of study designs and may hamper the ability of health authorities to assess the applicability of study findings [98]. Bushmeat surveys were generally short, with a mean data collection period of about two months, and three-quarters focused on a single type of study site. Similar design limitations have been noted in biodiversity-oriented bushmeat surveys [54], where experimental protocols fail to capture the complexity and dynamics of the bushmeat trade chain. In health surveys, appropriate temporal and spatial sampling scales can participate in an accurate assessment of zoonotic risk dynamics along the chain of complex interactions characterizing the bushmeat trade network [16,99]. Most bushmeat health risk surveys were conducted at the national level, with only 5.4% addressing the international scale, while recent outbreaks such as Mpox highlight the global public health relevance of the BT in a globally interconnected world [76,100]. Health risk assessments primarily targeted human communities, often through interviews on risk perceptions and behaviors (e.g., [101–103]). However, only one-third of these surveys examined bushmeat species, and less than 5% covered human-wildlife interactions, despite the critical role of host biology and human-animal interfaces in characterizing and anticipating zoonotic spillovers [104,105]. Moreover, direct investigations of zoonotic pathogens were relatively scarce, with interviews being the dominant survey method. This limited focus on pathogen detection may constitute an additional barrier to comprehensive zoonotic risk assessment in African tropical rainforests, given that it is a fundamental component of health risk quantification [106].

The publication delays in scientific research, as seen in health risk surveys related to the bushmeat (median = 2 years post-survey), along with the time lag between zoonotic spillovers and their publication (mean = 3.8 years), and the low proportion of studies conducted in direct response to outbreaks, highlight a key limitation: peer-reviewed research is not an immediate tool for epidemic or pandemic response. Instead, it might be considered as serving to analyze the processes and impacts of zoonotic outbreaks and potentially anticipate future risks (e.g., [107,108]). This is especially relevant in recent years, as researchers have increasingly relied on historical data to assess long-term trends or address emerging questions—such as the impact of zoonotic diseases in a post-pandemic context (e.g., [107,109,110]).

Despite a noticeable rise in scientific output since 2017 and –more globally– a bias of the bushmeat health risk surveys towards the African continent [62], we could not identify any study providing a probabilistic assessment of zoonotic spillover. The overwhelming majority of health surveys have concentrated on identifying potential hazards associated with bushmeat consumption and trade, rather than evaluating the likelihood of these risks occurring. Moreover, nearly half of the studies reviewed did not provide specific recommendations for mitigating health risks, despite widespread recognition of the human health threats posed by bushmeat practices (e.g., [42,111,112]). In contrast, the transmission of zoonotic diseases from domestic animals and livestock (such as cattle, pigs, poultry, dogs, cats, goats, etc.) to humans is a relatively well-studied topic (e.g., [113–116]), particularly in the context of livestock intensification and human-animal interactions [117,118]. Several studies have quantified these risks, using probabilistic methods (e.g., [119–122]).

Bushmeat species can host a wide array of pathogens—including viruses, bacteria, and parasites—whose presence is influenced by a multitude of factors and evolves over time and space, necessitating long-term monitoring for a comprehensive understanding [123]. Although the advent of metagenomics has enabled untargeted screening of entire pathogen communities, health-directed insights remain limited, as many studies lack essential data such as pathogen prevalence and host range—both of which are central to accurate zoonotic risk assessment [104]. Compounding this issue is a broader, global gap in knowledge regarding the actual consequences of exposure to many of the identified pathogens [42], and how the destructive process of unregulated hunting on ecosystem equilibrium may increase the risk of zoonotic spillovers [77,124]. As a result, trait- and network-based approaches—despite their widespread use in zoonotic risk quantification—are particularly difficult to apply in the African bushmeat context due to these persistent data deficiencies [see 104].

Bushmeat surveys are further constrained by limited access to key points along the supply chain—particularly the hunting stage, which often takes place in remote forested areas, and the opaque networks of middlemen that mediate trade [54]. These limitations hinder the ability to conduct comprehensive, system-wide exposure assessments along the bushmeat chain. The predominantly illegal and/or informal nature of the BT [79] fosters mistrust among stakeholders, complicating the implementation of surveys and regulatory measures [125]. Moreover, efforts to investigate and document bushmeat-related health risks may be perceived as threats to livelihoods and cultural traditions [28,126]. The lack of risk quantification is also rooted in divergent priorities among researchers, policymakers, donors, and local communities in many low-income countries [127,128]. In tropical Africa, public health funding is often directed toward well-established diseases (see [129]), leaving limited resources for research on bushmeat and other endemic zoonotic threats [18,130].

The failure to quantify health risks associated with bushmeat consumption and handling poses a significant public health challenge for African tropical rainforests —a region recognized as a hotspot for both BT and the emergence of zoonotic diseases (see [99]). In the absence of robust risk assessments, healthcare systems may remain ill-equipped to anticipate and respond to outbreaks, with populations risking repeated exposure to high-priority zoonotic diseases [13,131]. Although health risks can also be mitigated through precautionary, qualitative, and evidence-informed approaches that focus on exposure reduction and adaptive management [132–134], inaccurate anticipation of zoonotic risks can have serious repercussions for the food security and economic stability of rural communities and bushmeat stakeholders, who may lose a critical livelihood resource while also facing stigma during outbreaks and trade bans (e.g., [32, 135]).

### Avenues for reinforcing health risk assessment in the African bushmeat trade

Although progress has been made in predicting zoonotic spillovers and the epidemic potential of pathogens through advances in zoonotic risk assessment protocols [136], quantitative evaluations of such risks remain virtually absent from the bushmeat literature. This may be due to the fact that available approaches, such as Quantitative Risk Assessment Model (QRAM), require knowledge on parameters such as pathogen prevalence, dose-response relationships and frequency of exposure [137], that are difficult to measure in the African bushmeat context.

Because bushmeat research has largely focused on specific pathogens in post-epidemic, reactive investigations (see [83]), our understanding of pathogen diversity, distribution, and prevalence in bushmeat species remains limited [43]. However, advances in high-throughput sequencing now enable the broad, “universal” screening of microbiomes from virtually any RNA or DNA matrix [138,139]. This approach makes it possible to identify key indicators of spillover risk, such as the frequency of pathogen occurrence across multiple host species and pathogen load in specific tissues. With the increasing portability and affordability of these technologies, their application under tropical field conditions has become feasible [140,141]. The systematic integration of such approaches into bushmeat surveys would provide critical insights into health risks.

Modeling dose–response relationships in bushmeat species remains challenging due to the scarcity of experimental exposure data [142]. Although some efforts have been made, they have largely focused on a few major zoonotic agents (e.g., Ebola virus; [143]). The complexity in modeling such relationships also arises from the fact that response likely results from interplay between latent infections, host resistance, and pathogen passage histories (i.e., multiple infections), all of which relate to the ecology of the host species [144,145]. Improving risk characterization therefore depends on addressing critical knowledge gaps concerning the natural history of bushmeat species and their interactions in the wild.

Given the cryptic and unregulated nature of the bushmeat trade, estimating the frequency of exposure to health risks remains challenging. A necessary first step toward this goal would be to map the complexity of the bushmeat supply chain and adapt a Hazard Analysis and Critical Control Points framework to this informal network, thereby identifying Critical Control Points (CCPs) at which exposure frequency can be quantified (e.g., [146]). These CCPs should also account for food-borne disease risks occurring at the consumer end of the chain, which remain poorly characterized in the bushmeat context [30]. Moreover, understanding the dynamics of the bushmeat trade is essential, as accurate risk quantification depends on knowledge of the most frequently traded species (which maximize contacts with humans; [147]), the seasonality of hunting volumes (which may interact with pathogen transmission cycles; [148]), and the configuration of market stalls and traders’ practices (which can facilitate interspecies spillover, particularly when live animals are displayed; [149]).

Recently, Fourchault et al. [150] developed a categorical risk-scoring framework to assess the likelihood of zoonotic pathogen spillover in traditional medicine markets in Africa. Although this semi-quantitative approach is effective for identifying critical control points, guiding local intervention strategies, and operating under data-scarce conditions (e.g., [151]), its scoring weights rely heavily on literature-based data and strong a priori assumptions. As a result, it may oversimplify complex host–pathogen–environment interactions and does not provide a direct quantification of health risk.

Based on our review, we conclude that the capacity for health risk assessment in the context of the African BT remains far from being achieved. Substantial efforts are needed to unravel the structure and seasonal dynamics of bushmeat supply chains and to identify biodiversity and zoonotic pathogen trade hotspots. Importantly, special attention should be given to bridging the “species barrier” that still separates human and non-human health studies (see [152]). We argue that adopting a One Health framework for health surveillance of the BT—integrating simultaneous monitoring of wildlife hosts, vectors, and humans at the animal–human interface—is essential for advancing quantitative risk assessment. Given that wildlife trade in general, and the BT in particular, represent likely candidates for the next “Disease X” [153], we urge scientists, practitioners, and intergovernmental agencies to adopt this interdisciplinary, cross-sectoral approach in the near future. Ultimately, only the formal recognition of the bushmeat supply chain by national authorities, followed by its structured regulation and oversight, will make comprehensive and durable health risk quantification possible.

## Supporting information

S1 Table PRISMA 2020 checklist for reporting systematic reviews. This table summarizes compliance with the PRISMA Statement guidelines. Each item corresponds to recommended reporting standards, with locations where the criteria are addressed in the manuscript.
(DOCX)

S2 Table Database of all records identified in the literature search, including excluded records and reasons for exclusion. (XLSB)

S3 Table Summary of the 129 peer-reviewed articles included in the study. Latitude and longitude are expressed in decimal degrees.
(XLSX)

S4 Table List of the 139 bushmeat taxa recorded as hosts of microbes and other organisms, mostly pathogens. (DOCX)

S1 Appendix Definition of selected variables and descriptors extracted from the systematic review (see S3 Table). (DOCX)
