# Supplementary material for: Systematic review of health risk assessment in Africa’s bushmeat trade: Are there any risks assessed?
Source: PLoS Negl Trop Dis. 2026 May 18;20(5):e0014308. doi: 10.1371/journal.pntd.0014308 (PMC13197069; doi:10.1371/journal.pntd.0014308)
Supplement: S4 Table — (DOCX) [file pntd.0014308.s004.docx]

| **Number of studies** | **Genus** | **Species** | **Order** | **Class** | **Distribution range** | **IUCN RL** |
| --- | --- | --- | --- | --- | --- | --- |
| 1 | *Galloanserae* | *_* | Anseriformes, Galliformes | Aves | / | / |
| 1 | *Francolinus* | *bicalcarutus* | Galliformes | Aves | West and Central Africa | LC |
| 1 | *Numida* | *meleagris* | Galliformes | Aves | Sub-Saharian Africa | LC |
| 1 | *Aepyceros* | *melampus* | Artiodactyla | Mammalia | East Africa | LC |
| 1 | *Alcelaphus* | *buselaphus majeur* | Artiodactyla | Mammalia | West and Central Africa | LC |
| 1 | Antelopes | *_* | Artiodactyla | Mammalia | / | / |
| 1 | *Cephalophus* | *callipygus* | Artiodactyla | Mammalia | Central Africa | LC |
| 1 | *Cephalophus* | *dorsalis* | Artiodactyla | Mammalia | Central Africa | LC |
| 1 | *Cephalophus* | *niger* | Artiodactyla | Mammalia | West Africa | LC |
| 1 | *Cephalophus* | *nigrifrons* | Artiodactyla | Mammalia | Central Africa | LC |
| 2 | *Cephalophus* | *rufilatus* | Artiodactyla | Mammalia | West and Central Africa | LC |
| 5 | *Cephalophus* | spp. | Artiodactyla | Mammalia | NA | / |
| 1 | *Connochaetes* | sp. | Artiodactyla | Mammalia | Sub-Saharian Africa | LC |
| 2 | *Connochaetes* | *taurinus* | Artiodactyla | Mammalia | East and Southern Africa | LC |
| 1 | *Eudorcas* | *thomsonii* | Artiodactyla | Mammalia | East Africa | LC |
| 2 | *Kobus* | *ellipsiprymnus* | Artiodactyla | Mammalia | Sub-Saharian Africa | LC |
| 1 | *Kobus* | sp. | Artiodactyla | Mammalia | Africa | / |
| 1 | *Madoqua* | *kirkii* | Artiodactyla | Mammalia | East and Southern Africa | LC |
| 1 | *Neotragus* | *pygmaeus* | Artiodactyla | Mammalia | West Africa | LC |
| 1 | *Phacochoerus* | *aethiopicus* | Artiodactyla | Mammalia | East and Central Africa | LC |
| 2 | *Phacochoerus* | *africanus* | Artiodactyla | Mammalia | Sub-Saharian Africa | LC |
| 3 | *Philantomba* | *maxwelli* | Artiodactyla | Mammalia | West Africa | LC |
| 2 | *Philantomba* | *monticola* | Artiodactyla | Mammalia | Central Africa | LC |
| 3 | *Philantomba* | *walteri* | Artiodactyla | Mammalia | West and East Africa | DD |
| 2 | *Potamochoerus* | *porcus* | Artiodactyla | Mammalia | West and Central Africa | LC |
| 4 | *Sylvicapra* | *grimmia* | Artiodactyla | Mammalia | Sub-Saharian Africa | LC |
| 1 | *Taurotragus* | *derbianus* | Artiodactyla | Mammalia | West and Central Africa | VU |
| 3 | *Tragelaphus* | *scriptus* | Artiodactyla | Mammalia | Sub-Saharian Africa | LC |
| 1 | *Tragelaphus* | *spekii* | Artiodactyla | Mammalia | West, Central and East Africa | LC |
| 1 | *Tragelaphus* | *strepsiceros* | Artiodactyla | Mammalia | Sub-Saharian Africa | LC |
| 1 | *Tragelaphus* | *sylvaticus* | Artiodactyla | Mammalia | Sub-Saharian Africa | LC |
| 1 | *Caracal* | *aurata* | Carnivora | Mammalia | West and Central Africa | VU |
| 4 | *Civettictis* | *civetta* | Carnivora | Mammalia | Sub-Saharian Africa | LC |
| 2 | *Crossarchus* | *platycephalus* | Carnivora | Mammalia | West and Central Africa | LC |
| 2 | *Felis* | *silvestris* | Carnivora | Mammalia | West and Central Africa | LC |
| 2 | *Genetta* | *maculata* | Carnivora | Mammalia | Sub-Saharian Africa | LC |
| 1 | *Genetta* | spp. | Carnivora | Mammalia | Sub-Saharian Africa | / |
| 2 | *Herpestes* | *ichneumon* | Carnivora | Mammalia | Africa | LC |
| 4 | *Nandinia* | *binotata* | Carnivora | Mammalia | Sub-Saharian Africa | LC |
| 2 | *Chaerephon* | spp. | Chiroptera | Mammalia | NA | / |
| 1 | *Doryrhina* | *cyclops* | Chiroptera | Mammalia | west and Central Africa | LC |
| 6 | *Eidolon* | *helvum* | Chiroptera | Mammalia | Sub-Saharian Africa | NT |
| 5 | *Epomophorus* | *gambianus* | Chiroptera | Mammalia | West, Central and East Africa | LC |
| 5 | *Epomops* | *buettikoferi* | Chiroptera | Mammalia | West Africa | LC |
| 3 | *Epomops* | *franqueti* | Chiroptera | Mammalia | West and Central Africa | LC |
| 1 | *Hipposideros* | *beatus* | Chiroptera | Mammalia | west and Central Africa | LC |
| 1 | *Hipposideros* | *fuliginosus* | Chiroptera | Mammalia | West and Central Africa | LC |
| 1 | *Hipposideros* | *gigas* | Chiroptera | Mammalia | West and Central Africa | LC |
| 1 | *Hipposideros* | *jonesi* | Chiroptera | Mammalia | West Africa | NT |
| 1 | *Hipposideros* | *ruber/caffer* | Chiroptera | Mammalia | Sub-Saharian Africa | LC |
| 1 | *Hipposideros* | *abae* | Chiroptera | Mammalia | West, Central and East Africa | LC |
| 2 | *Hipposideros* | spp. | Chiroptera | Mammalia | Sub-Saharian Africa | / |
| 2 | *Hypsignathus* | *monstrosus* | Chiroptera | Mammalia | West and Central Africa | LC |
| 2 | *Lissonycteris* | *angolensis* | Chiroptera | Mammalia | Central Africa | LC |
| 2 | *Micropteropus* | *pusillus* | Chiroptera | Mammalia | West, Central and East Africa | LC |
| 5 | *Mops* | *condylurus* | Chiroptera | Mammalia | Sub-Saharian Africa | LC |
| 2 | *Nanonycteris* | *torquata* | Chiroptera | Mammalia | Sub-Saharian Africa | LC |
| 2 | *Nycteris* | spp. | Chiroptera | Mammalia | NA | / |
| 2 | *Rhinolophus* | spp. | Chiroptera | Mammalia | NA | / |
| 1 | *Rousettus* | *aegyptiacus* | Chiroptera | Mammalia | Sub-Saharian Africa | LC |
| 2 | *Scotophilus* | *leucogaster* | Chiroptera | Mammalia | Sub-Saharian Africa | LC |
| 3 | *Atelerix* | *albiventris* | Erinaceomorpha | Mammalia | West, Central and East Africa | LC |
| 1 | *Dendrohyrax* | *dorsalis* | Hyracoidea | Mammalia | west and central Africa | LC |
| 1 | *Dendrohyrax* | *interfluvialis* | Hyracoidea | Mammalia | West and Central Africa | DD |
| 1 | *Lepus* | *microtus* | Lagomorpha | Mammalia | Africa | LC |
| 1 | *Lepus* | spp. | Lagomorpha | Mammalia | NA | / |
| 1 | *Lepus* | *victoriae* | Lagomorpha | Mammalia | Africa | LC |
| 3 | *Oryctolagus* | *cuniculus* | Lagomorpha | Mammalia | Sub-Saharian Africa | EN |
| 1 | *Petrodromus* | *tetradactylus* | Macroscelidea | Mammalia | Central, East and Southern Africa | LC |
| 7 | *Phataginus* | *tricuspis* | Pholidota | Mammalia | West and Central Africa | EN |
| 1 | *Phataginus* | spp. | Pholidota | Mammalia | NA | / |
| 1 | *Phataginus* | *tetradactyla* | Pholidota | Mammalia | West and Central Africa | VU |
| 1 | *Smutsia* | *gigantea* | Pholidota | Mammalia | West and Central Africa | EN |
| 1 | *Allenopithecus* | *nigroviridis* | Primates | Mammalia | Central Africa | LC |
| 3 | *Cercocebus* | *agilis* | Primates | Mammalia | Central Africa | LC |
| 1 | *Cercocebus* | *atys* | Primates | Mammalia | West Africa | VU |
| 8 | *Cercocebus* | *torquatus* | Primates | Mammalia | West and Central Africa | EN |
| 1 | *Cercopithecus* | *ascanius* | Primates | Mammalia | West and Central Africa | LC |
| 1 | *Cercopithecus* | *ascanius schmidti* | Primates | Mammalia | East and Central Africa | LC |
| 1 | *Cercopithecus* | *ascanius whitesidei* | Primates | Mammalia | Central Africa | LC |
| 1 | *Cercopithecus* | *campbelli* | Primates | Mammalia | West and Central Africa | NT |
| 9 | *Cercopithecus* | *cephus* | Primates | Mammalia | Central Africa | LC |
| 1 | *Cercopithecus* | *diana* | Primates | Mammalia | West Africa | EN |
| 2 | *Cercopithecus* | *erythrogaster* | Primates | Mammalia | West and Central Africa | EN |
| 1 | *Cercopithecus* | *hamlyni* | Primates | Mammalia | East and Central Africa | VU |
| 1 | *Cercopithecus* | *lhoesti* | Primates | Mammalia | East and Central Africa | VU |
| 1 | *Cercopithecus* | *mitis* | Primates | Mammalia | Central, East and Southern Africa | LC |
| 8 | *Cercopithecus* | *mona* | Primates | Mammalia | West and Central Africa | NT |
| 9 | *Cercopithecus* | *neglectus* | Primates | Mammalia | East and Central Africa | LC |
| 10 | *Cercopithecus* | *nictitans* | Primates | Mammalia | Central Africa | NT |
| 1 | *Cercopithecus* | *petaurista* | Primates | Mammalia | West and Central Africa | NT |
| 5 | *Cercopithecus* | *pogonias* | Primates | Mammalia | West and Central Africa | NT |
| 1 | *Cercopithecus* | *preussi* | Primates | Mammalia | West and Central Africa | EN |
| 1 | *Cercopithecus* | *solatus* | Primates | Mammalia | Gabon | NT |
| 3 | *Cercopithecus* | spp. | Primates | Mammalia | Sub-Saharian Africa | / |
| 1 | *Cercopithecus* | *wolfi* | Primates | Mammalia | Central Africa | NT |
| 4 | *Chlorocebus* | *tantalus* | Primates | Mammalia | West, Central and East Africa | LC |
| 1 | *Chlorocebus* | *aethiops* | Primates | Mammalia | East and Central Africa | LC |
| 7 | *Colobus* | *guereza* | Primates | Mammalia | West, Central and East Africa | LC |
| 3 | *Erythrocebus* | *patas* | Primates | Mammalia | West and Central Africa | NT |
| 1 | Galagidae | *_* | Primates | Mammalia | West and Central Africa | / |
| 2 | *Gorilla* | *gorilla* | Primates | Mammalia | Central Africa | CR |
| 4 | *Lophocebus* | *albigena* | Primates | Mammalia | Central Africa | VU |
| 1 | *Lophocebus* | *aterrimus* | Primates | Mammalia | Central Africa | VU |
| 1 | *Mandrillus* | *leucophaeus* | Primates | Mammalia | west and Central Africa | EN |
| 8 | *Mandrillus* | *sphinx* | Primates | Mammalia | Central Africa | VU |
| 6 | *Miopithecus* | *ogouensis* | Primates | Mammalia | Central Africa | NT |
| 1 | *Miopithecus* | *talapoin* | Primates | Mammalia | Central Africa | VU |
| 9 | *Pan* | *troglodytes* | Primates | Mammalia | West and Central Africa | EN |
| 3 | *Papio* | *anubis* | Primates | Mammalia | West, Central and East Africa | LC |
| 1 | *Perodicticus* | *potto* | Primates | Mammalia | West and Central Africa | NT |
| 1 | *Piliocolobus* | *badius* | Primates | Mammalia | West and Central Africa | EN |
| 1 | *Piliocolobus* | *tholloni* | Primates | Mammalia | Central Africa | VU |
| 1 | Unidentified primate | *_* | Primates | Mammalia | / | / |
| 1 | *Loxodonta* | *africana* | Proboscidea | Mammalia | Sub-Saharian Africa | EN |
| 1 | *Arvicanthis* | *niloticus* | Rodentia | Mammalia | Sub-Saharian Africa | LC |
| 4 | *Atherurus* | *africanus* | Rodentia | Mammalia | Central Africa | LC |
| 2 | *Cricetomys* | *emini* | Rodentia | Mammalia | Central Africa | LC |
| 2 | *Cricetomys* | spp. | Rodentia | Mammalia | Sub-Saharian Africa | / |
| 1 | *Cricetomys* | *gambianus* | Rodentia | Mammalia | Sub-Saharian Africa | LC |
| 1 | *Funisciurus* | spp. | Rodentia | Mammalia | Central Africa | / |
| 1 | *Graphiurus* | *lorraineus* | Rodentia | Mammalia | Central Africa | LC |
| 1 | *Heliosciurus* | spp. | Rodentia | Mammalia | West and Central Africa | / |
| 1 | *Helioscurius* | *rufobrachium* | Rodentia | Mammalia | West, Central and East Africa | LC |
| 2 | *Hystrix* | *cristata* | Rodentia | Mammalia | Sub-Saharian Africa | LC |
| 1 | *Mops* | *condylurus* | Rodentia | Mammalia | Sub-Saharian Africa | LC |
| 1 | *Oenomys* | *hypoxanthus* | Rodentia | Mammalia | West, Central and East Africa | LC |
| 1 | *Protoxerus* | *stangeri* | Rodentia | Mammalia | West, Central and East Africa | LC |
| 1 | *Rattus* | *fuscipes* | Rodentia | Mammalia | West Africa | LC |
| 7 | *Thryonomys* | *swinderianus* | Rodentia | Mammalia | Sub-Saharian Africa | LC |
| 3 | *Xerus* | *erythropus* | Rodentia | Mammalia | Sub-Saharian Africa | LC |
| 1 | *Osteolamus* | spp. | Crocodylia | Reptilia | Central Africa | / |
| 1 | *Varanus* | *exanthematicus* | Crocodylia | Reptilia | Sub-Saharian Africa | LC |
| 1 | *Varanus* | *niloticus* | Crocodylia | Reptilia | Sub-Saharian Africa | LC |
| 1 | *Varanus* | *ornatus* | Crocodylia | Reptilia | Sub-Saharian Africa | LC |
| 1 | *Bitis* | spp. | Squamata | Reptilia | Sub-Saharian Africa | / |
| 1 | *Python* | *sebae* | Squamata | Reptilia | West, Central and East Africa | NT |
| 1 | *Python* | sp. | Squamata | Reptilia | West and Central Africa | NT |
