# Supplementary material for: Systematic review of health risk assessment in Africa’s bushmeat trade: Are there any risks assessed?
Source: PLoS Negl Trop Dis. 2026 May 18;20(5):e0014308. doi: 10.1371/journal.pntd.0014308 (PMC13197069; doi:10.1371/journal.pntd.0014308)
Supplement: S1 Appendix — (DOCX) [file pntd.0014308.s005.docx]

**Study context**

- **Field** – the main scientific fields to which the publications were related. Including public health surveillance (systematic collection, analysis and interpretation of health-related data), epidemiology (determinants, occurrence, and distribution of health and disease) and ethnobiology (interactions between humans and environment in the cultural context).
- **Scale –** the geographical scale at which the research is carried. Including international, national or regional.
- **Type of study site –** the type of site(s) surveyed. Including forest and protected areas; Hospitals; Households and schools; Rural communities; Rural bushmeat markets; Urban communities; Urban bushmeat markets.
- **Period –** start to end year of the study.

**Study design**

- **Risk assessment method** – the methods used by researchers to assess health risks related to bushmeat consumption and handling. Including: Biological tests (biochemical analysis of faecal, oral or blood samples, not involving PCR or sequencing); Interviews; Observations of risks practices; DNA-typing (screening [e.g., PCR] and sequencing of DNA/RNA material); Reviews.
- **Target actors** – the survey focus. Including humans (Households, Health workers, Zookeepers…) and bushmeat species (*Cercopithecus nictitans, Cercopithecus mona, Atherurus africanus*…)
- **Risks quantified?** – did the study conduct an actual estimate of the health risks, through the establishment of a probability, a score or any other conclusion in risk weighting?
- **Survey effort** – number of days used for data collection.
